# Complete removal of intraspinal extradural mass with unilateral biportal endoscopy

**DOI:** 10.3389/fsurg.2022.1033856

**Published:** 2022-11-11

**Authors:** Tao Wang, Hang Yu, Shi-bin Zhao, Bin Zhu, Lei Chen, Jue-hua Jing, Da-sheng Tian

**Affiliations:** Department of Orthopedics, Spine Surgery, Second Hospital of Anhui Medical University, Hefei, China

**Keywords:** unilateral biportal endoscopy (UBE), endoscopic spine surgery, extradural mass, cyst, hemangioma

## Abstract

**Introduction:**

Unilateral biportal endoscopic (UBE) technique can easily decompress the bony spinal canal and accommodate all open surgical instruments under endoscopic guidance. However, indications and reports of this technique have been limited to degenerative and infectious diseases.

**Methods:**

We used the UBE technique for the decompression and removal of extradural mass lesions in five patients. Under endoscopic guidance, a unilateral approach was used, and decompression and flavectomy were performed. After decompression, removal of the tumor was performed using various forceps. We evaluated the technical process of the procedure, the patient's pre- and postoperative symptoms, and operative radiology and pathologic results.

**Results:**

Postoperative pain and disability improved clinically for all patients. Four patients were confirmed as having an epidural cyst and one patient was diagnosed with hemangioma. During follow-up, no recurrence was observed.

**Conclusions:**

We successfully removed five extradural mass lesions using a biportal endoscopic posterior approach without complications. The biportal endoscopic approach may have advantages, such as minimizing trauma to the normal structures, magnified endoscopic view, and early recovery after the surgery. Biportal endoscopy may be used as an alternative surgical treatment for symptomatic intraspinal extradural benign lesions.

## Introduction

Extradural spinal masses stem from soft or bony tissues and can cause clinical symptoms related to axial destruction of the bony structure, as well as myelopathy and radiculopathy caused by spinal cord and nerve compression. Traditionally, open surgery was performed for an extradural mass. This method should split the paravertebral muscles, resect both laminae, and be followed by a pedicle screw fixation. This definitely increased patients' psychological and economic burdens. Later, uniportal endoscopy was reported to treat epidural arachnoid cysts (1), schwannoma (2), and even metastasis tumors (3) as a minimally invasive technique.

**Table 1:** The details of five extradural mass patients who received UBE

Patient No.	Gender	Age	Location	Pathology
1	M	37	T2–T3, left–right	Hemangioma
2	F	74	T12–L2, left–right	Epidural cyst
3	F	63	L4/5, left	Fact joint cyst
4	F	79	L4/5, left	Cyst
5	M	40	L5S1, left	Benign cyst

Unilateral biportal endoscopy (UBE) is an emerging technique among various minimally invasive spinal surgery options with free handling of the instruments under a magnified clear view. For spinal degenerative (4–6), trauma (7), and infectious disease (8), compared with conventional methods, UBE has achieved favorable clinical outcomes with several advantages such as minimal blood loss, reduced length of hospital stay, and reduced postoperative pain. Kim et al. demonstrate the UBE technique for an aneurysmal bone cyst biopsy and removal in a 72-year-old female patient with dramatic improvement of symptoms (9). However, the UBE technique for an extradural mass had limited reports.

In this study, we describe five cases that clarify how to use UBE to completely remove extradural mass lesions with obvious improvement of symptoms (Table 1).

## Methods

The patients underwent surgery under general anesthesia in the prone position. The number of incisions was made according to the extent of lesion involvement. Under image intensification, paramedian skin incisions were created along the medial pedicle line for ipsilateral spinal canal and foraminal decompression. After serial dilation, an endoscopic portal and a work portal were created. After soft-tissue dissection using a radiofrequency (RF) probe, the entire ipsilateral lamina, facet joint, and interlaminar window were exposed. First, a cranial laminotomy was performed along the inferior border of the upper lamina and the medial part of the inferior articular process using a 3.5-mm-diameter endoscopic diamond drill. Drilling was extended cranially until the proximal free margin of the ligamentum flavum was exposed. Therefore, the laminotomy was extended until the adhesive tissue faded, and the free epidural space was confirmed. After broad drilling of the spinous process base, contralateral sublaminar bony drilling was performed by crossing the midline to expose the medial end of the extradural mass. Subsequently, caudal laminotomy was performed by drilling the medial superior articular process and the superior part of the lower lamina until the distal free end of the ligamentum flavum and free epidural space was exposed. The ligamentum flavum was detached from the epidural adhesion tissues and bony margins using dissectors and punches. After removing the ligamentum flavum, the mass was exposed. The dissecting plane between the dura and mass was meticulously created using a nerve hook. Careful dissection was continued along the dissection plane until the mass was entirely detached from the dura. Finally, the extradural mass was removed en bloc. The skin wounds were closed by inserting a drainage catheter through the working portal.

## Case presentation

### Case 1

**Figure 1:**
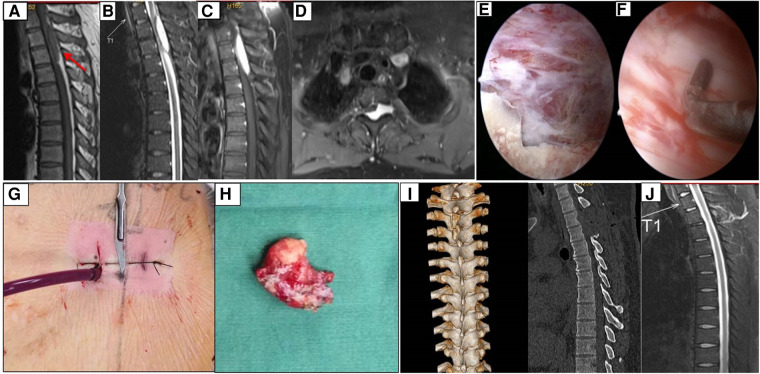
Case 1, extradural hemangioma was en bloc removed by unilateral biportal endoscopy. (**A–C**) The mass was hypo-intensity on T1, hyper-intensity on T2, and homogenous enhancement after Gd administration. (**D**) The axial image showed the mass involved the left foraminal, crossed the midline, and spinal cord was obviously compressed. (**E,F**) Endoscopic findings revealed a reddish mass compress the dural sac, and the dural sac was intact after totally removal of the mass. (**G,H**) The incisions and the mass resected. (**I**) Reconstructed CT showed hemi-laminectomy from T2 to T3, and the fact joint and spinal process was reserved. (**J**) MRI showed the spinal cord was decompressed and no recurrence was observed after six months follow-up.

A 47-year-old man presented with a 3-month history of insidious-onset and progressively aggravated motor weakness in his lower extremities. In addition, he had a chest band feeling during the most recent month. A neurological examination revealed no obvious sensory abnormalities, and the motor power of his lower extremities decreased to grade 4 (out of 5). He showed a clumsy and staggered gait after 5 min of walking. Preoperative magnetic resonance imaging (MRI) showed a large intraspinal extradural mass, extending from the left foraminal area at the T2–3 level, across the midline to the contralateral (Figure 1). The mass showed hypo-intensity on T1-weighted image, hyper-intensity on T2-weighted image, and showed obvious homogenous enhancement on T1 fat suppression contrast-enhanced image. This extradural mass compressed the thoracic spinal cord significantly and was preoperatively suspected to be a hemangioma. We performed a posterior laminotomy with mass removal using a biportal endoscopic approach.

### Case 2

A 74-year-old woman complained of low back pain that radiated to the anterolateral thigh and motor weakness in both her legs for 3 months. No obvious changes were observed in her bowel and bladder functions. The strength of the bilateral quadriceps muscle was 4 out of 5; all muscle strength below the knees was intact. A sensory examination showed a slight decrease to pinprick over the anterior thigh below the groin corresponding to the second and third lumbar dermatomes.

**Figure 2:**
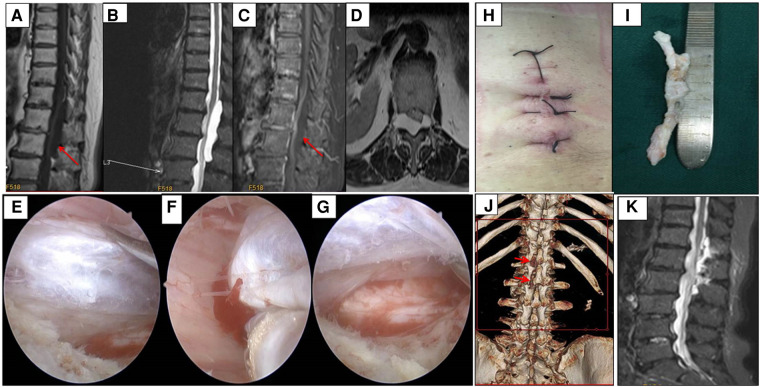
Case 2, epidural cyst was excised by unilateral biportal endoscopy. (**A–C**) The mass was hypo-intensity on T1, hyper-intensity on T2, and no enhancement after Gd administration. (**D**) The axial image showed the mass involved the right foraminal, crossed the midline, and compressed the spinal cord. (**E–G**) Endoscopic findings revealed a white mass compress the dural sac, and the dural sac was intact after the mass was removed. (**H,I**) The incisions and the gross specimen resected. (**J**) Reconstructed CT showed hemi-laminectomy from L1 to L2, and the fact joint and spinal process was reserved. (**K**) MRI showed the spinal cord was decompressed and no recurrence was observed 6 months later.

Preoperative T2-weighted MRI showed a large intraspinal extradural cystic mass, extending from the right foraminal area, across the midline to the contralateral at the T12–L2 level. Peripheral rim enhancement of the cystic mass was observed on T1-weighted contrast-enhanced MRI (Figure 2). The mass was deemed an epidural cystic mass before surgery. A unilateral biportal endoscopic approach was performed through posterior laminectomy with cyst removal.

## Results

Postoperatively, neurological deficits, including sensory, motor weakness, and back pain, improved in all patients. The hospital stay of all patients was in the range of 7–10 days. There was no recurrence of symptoms in either patient during the 6–12 months of follow-up. A pathological examination of surgical specimens revealed cysts in four patients and a hemangioma in one patient. No fixation was performed in either patient. Postoperative MRI showed a sufficiently decompressed spinal canal and foramen after the complete removal of the extradural mass. Postoperative CT images revealed a hemi-laminotomy of the lamina while preserving the spinous process and the facet joint.

## Discussion

Intraspinal extradural benign mass lesions, such as hemangioma and epidural cyst, are not uncommon. In the past, the most commonly used treatment is surgical removal by laminectomy via open surgery. Large lesions extending over two or more vertebral segments require extensive laminectomy and subsequent fixation usually performed in order to avoid anterior subluxation or kyphosis of the spine. In addition, open surgery has the disadvantages of being a long operation and having a long hospitalization time, large surgical trauma, more intraoperative bleeding, and slow recovery. In order to reduce the injury to the vertebral lamina and posterior muscles, less invasive procedures should be pursued for benign masses involving the spinal canal. Percutaneous uniportal endoscopy, which is used for disectomy, was reported to treat extradural arachnoid cysts, schwannomas, hematomas, and even metastatic tumor, through a transforaminal or interlaminar approach (1–3, 10, 11). However, due to the coaxial observation channel and work channel, it has the disadvantages of being an inconvenient operation and having limited surgical vision, and their application in bilateral decompression is limited (12).

Unilateral biportal endoscopy, which separates the observation and operation channels, has the advantages of being a flexible operation, having clear surgical vision, and being easy for bilateral decompression. The literature on the biportal technique is mostly limited to treatments for degenerative disease, including spinal stenosis decompression, herniated disc removal, and interbody fusion for instability (13). Since 2018, our spine center has used UBE to treat spinal degenerative disease, including multi-segment decompression and bilateral decompression. Based on previous experience, we have used UBE to treat intraspinal extradural benign mass lesions and achieved good results.

To treat intraspinal extradural mass lesions with the UBE technique, there are several areas of concern. First, what kind of extradural mass can be treated with UBE? In our case series, all masses were mainly located at the dorsal part of the spinal cord, and the mass should not extend to both extraforaminal areas. UBE is also a candidate for anterolateral masses located at the lumbar spine; however, for anterolateral masses in the cervical or thoracic spine, UBE is not the first choice, because dragging the spinal cord or nerve root is dangerous. The pathology of our mass is benign. So far, extradural masses treated with UBE have been mostly benign; a primary malignant tumor was not illustrated (14, 15). This technique may be an alternative decompression method for patients with metastatic tumors who would be unable to tolerate radical surgery. Next, on which side should the incision be made? If the mass did not cross the midline of the spinal cord, the incision was made at the ipsilateral side where the mass was located. If the mass crossed the midline, the incision was made at the side on which compression was mild. Third, how to design incisions? The extent of incisions should be in line with preoperative imaging data, and the work instruments should easily expose the free end of the mass. The number of incisions depended on the disc level of the mass involved: the number of incisions equal to the disc levels +1. For example, in case 2, the upper and lower borders of the mass were T12 and L2, respectively, and involved two disc levels (T12/L1, L1/2); three incisions were made: the upper and lower were transverse lines and the middle was a vertical line. Fourth, if bleeding occurs during the procedure, bone bleeding should be controlled using an RF coagulator or bone wax, while soft-tissue bleeding should be stopped using an RF coagulator with low frequency. If diffuse and multifocal bleeding occur or bleeding focus is not clear, brain cotton compression is a useful option for solving the problem. Fifth, en bloc resection is recommended. As a piecemeal removal pattern prevents identification of the dissecting plane between the dura and the mass, repetitive manipulation during piecemeal resection increases the risk of dural injury (16). In addition, en bloc resection is beneficial for complete mass removal to decrease the possibility of recurrence. Sixth, if incidental durotomy occurs during endoscopic operation, size should be evaluated first. When the tear size is small, we use brain cotton to compress the crevasse; when the size is big, UBE should be converted to open microscopic surgery for complete dural repair because the continuous saline infusion during UBE could increase the intracranial pressure (17). Fortunately, in our five cases, a durotomy did not take place. Last, but not least, UBE for extradural masses has a steep learning curve. Surgeons should have sufficient experience in the endoscopic posterior approach, from the cervical spine to the lumbar spine.

Although the UBE technique for intraspinal extradural benign masses has achieved good results in our cases and has impressive advantages, this technique should be considered in select patients. Large masses with obvious neurological deficits should be considered for open surgery. If facet joints are broken or segmental instability is found on preoperative radiographic images, stabilization is recommended to avoid kyphosis.

## Data Availability

The datasets presented in this article are not readily available. Requests to access the datasets should be directed to Wang Tao (wangtao1908@163.com).
